# Goniothalamin-induced oxidative stress, DNA damage and apoptosis via caspase-2 independent and Bcl-2 independent pathways in Jurkat T-cells

**DOI:** 10.1016/j.toxlet.2009.12.010

**Published:** 2010-03-01

**Authors:** S.H. Inayat-Hussain, K.M. Chan, N.F. Rajab, L.B. Din, S.C. Chow, A. Kizilors, F. Farzaneh, G.T. Williams

**Affiliations:** a Toxicology and Biocompatibility Laboratory, Faculty of Allied Health Sciences, Universiti Kebangsaan Malaysia, Jalan Raja Muda Abdul Aziz, 50300 Kuala Lumpur, Malaysia; b UKM Medical Molecular Biology Institute, Universiti Kebangsaan Malaysia, Bandar Tun Razak, 56000 Kuala Lumpur, Malaysia; c Faculty of Science and Technology, Universiti Kebangsaan Malaysia, Bangi 43600 Selangor DE, Malaysia; d School of Science, Monash University, Sunway Campus, Malaysia; e King's College London, Department of Haematological Medicine, The Rayne Institute, 123, Coldharbour Lane, London, SE5 9NU, UK; f Institute for Science and Technology in Medicine, School of Life Sciences, Keele University, Keele ST5 5AZ, UK

**Keywords:** Goniothalamin, DNA damage, Oxidative stress, Caspase-2, Bcl-2

## Abstract

Goniothalamin (GTN) isolated from *Goniothalamus* sp. has been demonstrated to induce apoptosis in a variety of cancer cell lines including Jurkat T leukemia cells. However, the mechanism of GTN-induced apoptosis upstream of mitochondria is still poorly defined. In this study, GTN caused a decrease in GSH with an elevation of reactive oxygen species as early as 30 min and DNA damage as assessed by Comet assay. Analysis using topoisomerase II processing of supercoiled pBR 322 DNA showed that GTN caused DNA damage via a topoisomerase II-independent pathway suggesting that cellular oxidative stress may contribute to genotoxicity. A 12-fold increase of caspase-2 activity was observed in GTN-treated Jurkat cells after 4 h treatment and this was confirmed using Western blotting. Although the caspase-2 inhibitor Z-VDVAD-FMK inhibited the proteolytic activity of caspase-2, apoptosis ensued confirming that caspase-2 activity was not crucial for GTN-induced apoptosis. However, GTN-induced apoptosis was completely abrogated by N-acetylcysteine further confirming the role of oxidative stress. Since cytochrome *c* release was observed as early as 1 h without any appreciable change in Bcl-2 protein expression, we further investigated whether overexpression of Bcl-2 confers resistance in GTN-induced cytotoxicity. Using a panel of Jurkat Bcl-2 transfectants, GTN cytotoxicity was not abrogated in these cells. In conclusion, GTN induces DNA damage and oxidative stress resulting in apoptosis which is independent of both caspase-2 and Bcl-2.

## 1 Introduction

Styryl-lactones are secondary metabolites isolated from *Goniothalamus* plant species. Currently, there are approximately 100 styryl-lactones either discovered from natural products or synthetic analogs. These compounds have been demonstrated to be cytotoxic with prefential killing of cancer cells (de Fátima et al., 2006). Goniothalamin (GTN) a plant styryl-lactone isolated from *Goniothalamus andersonii*, induces cytotoxicity in a variety of cancer cell lines including cervical (Hela), gastric (HGC-27), ovarian (Caov-3), kidney (786-0), breast carcinomas (MCF7, T47D and MDA-MB-231) and leukemia (HL-60, Jurkat and CEM-SS) (Ali et al., 1997; Inayat-Hussain et al., 1999, 2003; Rajab et al., 2005; Pihie et al., 1998; Alvin Lee and Azimahtol Hawariah, 2003; Teoh and Azimahtol Hawariah, 2000; de Fátima et al., 2006). The mechanism of GTN-induced cytotoxicity in human leukemia (HL-60 and Jurkat) and human breast cancer cells (MDA-MB-231) has been confirmed to occur via apoptosis (Chen et al., 2005; Inayat-Hussain et al., 1999, 2003).

It has been reported that cytotoxic stress either from DNA damage or mitochondrial impairment leads to apoptosis via the intrinsic pathway (Chan et al., 2006; Kang and Reynolds, 2009). The intrinsic pathway involves the release of proapoptotic proteins including cytochrome *c* from the inner membrane of mitochondria to the cytosol leading to activation of caspase-9 (Riedl and Salvesen, 2007). Numerous studies have demonstrated that the oncoprotein Bcl-2 can inhibit apoptosis by inhibiting the release of cytochrome *c* and can also modulate oxidant induced apoptosis (Schwartz and Hockenbery, 2006). Since the discovery of the caspase-9 apoptosome complex (Zou et al., 1999), more recent studies have shown that the initiator caspase-2 also forms a complex with RAIDD, a death receptor molecule and the p53 inducible death domain PIDD forming a PIDDosome complex (Tinel and Tschopp, 2004). Importantly, caspase-2 has been demonstrated in a variety of cell lines to be activated upstream of mitochondria in genotoxin-induced apoptosis. Cleavage of the proapoptotic Bcl-2 family member Bid by caspase-2 has been shown to be required for cytochrome *c* release suggesting a potentially crucial role for caspase-2.

Since goniothalamin has recently been demonstrated to induce DNA damage in vascular smooth muscle cells, HL-60 and CEM-SS leukemia cells (Rajab et al., 2005), the role of caspase-2 in goniothalamin-induced apoptosis was investigated in this study. Furthermore, the anti-apoptotic effects of Bcl-2 were further determined using a panel of Bcl-2-transfected Jurkat cells expressing varying level of Bcl-2 proteins (Johnson et al., 1999). Our results demonstrate that goniothalamin-induced DNA damage and ROS leads to apoptosis through caspase-2- and Bcl-2-independent pathways.

## 2 Materials and methods

### 2.1 Chemicals

Goniothalamin was obtained from *Goniothalamus andersonii* as described previously (Inayat-Hussain et al., 1999). All other chemicals were purchased from Sigma unless stated otherwise.

### 2.2 Cell culture

Jurkat T lymphoblastic leukemia cells (clone E6-1) were purchased from ATCC and maintained in RPMI-1640 medium (GIBCO) supplemented with 10% heat inactivated fetal calf serum (PAA), 2 mM l-glutamine and 1% pen/strep (PAA), at 37 °C in a 5% CO_2_ humidified incubator. All experiments were carried out using cells in logarithmic growth phase. Bcl-2 transfected cells were obtained as described previously (Johnson et al., 1999) and were maintained in Hygromycin B (Merck) supplemented medium to maintain the overexpression of Bcl-2.

### 2.3 Determination of GSH

GSH was quantified as described previously by Ellman (1959) with modifications. Briefly, 50 μM GTN-treated cells (1 × 10^6^ cells) were collected and washed with chilled PBS. The cells were pelleted at 220 × *g* for 5 min. Subsequently, the supernatant was discarded and pellet was mixed with 100 μl of ice cold lysis buffer (50 mM K_2_HPO_4_, 1 mM EDTA, pH 6.5 and 0.1% v/v Triton X-100). The cells were incubated on ice for 15 min with gentle tapping from time to time. The crude lysates were cleared by centrifugation at 10,000 × *g* for 15 min at 4 °C. At this point, the lysates were used immediately or stored at −80 °C for up to 2 days. Then, 50 μl of lysates and GSH standards (twofold dilution from 1.25 mM to 0 mM dissolved in reaction buffer [0.1 M Na_2_HPO_4_·7H_2_O and 1 mM EDTA, pH 6.5]) were pipetted into designated wells in 96-well plate. After adding 40 μl of reaction buffer (0.1 M Na_2_HPO_4_·7H_2_O and 1 mM EDTA, pH 8), 10 μl of 4 mg/ml DTNB in reaction buffer pH 8 was added to wells containing samples and standards. The plate was incubated for a further 15 min at 37 °C. Absorbance of each well was measured at 405 nm using microplate reader. The concentration of free thiols in the samples was calculated based on GSH standard and expressed as nmol/mg protein.

### 2.4 Flow cytometry assessment of reactive oxygen species

The reactive oxygen species (ROS) level was assessed as described previously by Li et al. (2002) with slight modification. Briefly, GTN-treated cells (1 × 10^6^ cells) were collected via centrifugation. The supernatant was discarded and the cells were resuspended with 1 ml pre-warmed FBS-free fresh medium and 1 μl of 10 mM hydroethidine (HE) (Molecular Probes) solution was added. The cell suspension containing 10 μM HE was incubated in the dark at 37 °C for 30 min. Subsequently, the cells were centrifuged at 220 × *g* for 5 min and were washed with 1 ml chilled PBS. The supernatant was discarded and 500 μl of cold PBS was mixed with the pellets. The stained cell suspension was transferred to flow tubes and analyzed with FACSCalibur Flow Cytometer (BD Bioscience) using FL2 channel on 10,000 cells.

### 2.5 Alkaline comet assay

The comet assay was carried out as described previously (Chan et al., 2006). Briefly, 500 μl GTN-treated cells (1 × 10^6^ cells/ml) were transferred to a tube for centrifugation (2500 rpm/5 min at 4 °C). The supernatant was removed and pellet was washed with Ca^2+^- and Mg^2+^-free PBS and re-centrifuged. The cell pellets were mixed thoroughly with 80 μl of 0.6% low melting agar. The mixture was then pipetted onto the hardened 0.6% normal melting agar as the first layer gel on the slide. Cover slips were placed to spread the mixture and slides were left on ice for low melting agar to solidify. Following the removal of the cover slips, the embedded cells were lysed in a buffer containing 2.5 M NaCl, 100 mM EDTA, 10 mM Tris and 1% Triton X-100 for 1 h at 4 °C. Slides were soaked in electrophoresis buffer solution for 20 min for DNA unwinding before electrophoresis at 300 mA, 25 V for 20 min. Subsequently, the slides were rinsed with neutralising buffer for 5 min and stained with 50 μl of 10 μg/ml ethidium bromide solution. Slides were left overnight at 4 °C before analysis, using a fluorescent microscope equipped with 590 nm filter. The DNA damage was assessed on 50 cells where tail moment (tail length multiplied by the fraction of DNA in the tail) was quantified using the Comet Assay III (Perceptive Instrument Ltd.).

### 2.6 Immunoblotting

GTN-treated cells (2 × 10^6^ cells/ml) were prepared for SDS–PAGE as previously described (Inayat-Hussain et al., 1999). Cellular proteins were resolved on 12% (caspases-2, -3 and Bcl-2) or 15% (cytochrome *c*) SDS–polyacrylamide gels under denaturing conditions and blotted onto a PVDF membrane (Amersham). Mouse monoclonal anti-caspase-2, rabbit polyclonal anti-caspase-3 and rabbit monoclonal anti-Bcl-2 antibody were purchased from Cell Signaling Technology Inc. For detection of cytochrome *c*, the cell pellet was resuspended in cytosol extraction buffer, and cytosolic extracts were prepared as described previously (Herrera et al., 2001). Mouse monoclonal anti-cytochrome *c* antibody was purchased from BD Pharmingen. Actin was detected for loading control using mouse monoclonal anti-actin antibody. Proteins were detected by Enhanced Chemi-Luminescence staining.

### 2.7 Assessment of apoptosis

GTN-induced Jurkat cells apoptosis was assessed based on the externalization of phosphatidylserine. Cells treated with GTN (1 × 10^6^ cells) were collected and washed once with PBS. In some experiments, the cells were pre-treated with 50 μM Z-VAD-FMK, Z-VDVAD-FMK or 10 mM N-acetylcysteine (NAC) for 1 h. The pellet was suspended with 100 μl annexin binding buffer (10 mM Hepes/NaOH pH 7.4, 150 mM NaCl, 5 mM KCl, 1 mM MgCl2·6H_2_O and 1.8 mM CaCl_2_·2H_2_O) and the cells were mixed with 1.5 μl annexin V-FITC (Invitrogen). Following 15 min incubation in dark, 10 μl propodium iodide (50 μg/ml) was added to cells and which were incubated for a further 2 min. Annexin binding buffer (400 μl) was added to the cells suspension and analyzed with FACSCalibur Flow Cytometer (BD Bioscience).

### 2.8 Determination of caspase-2 proteolytic activity

The caspase-2 proteolytic activity was detected using BioVision Caspase-2 Fluorometric kit based on specific fluorogenic substrates. Briefly, 50 μM GTN-treated cells (2 × 10^6^ cells) with and without 1 h pretreatment with 50 μM Z-VDVAD-FMK (Calbiochem) were collected by centrifugation at 200 × *g* for 5 min. Cells were washed with chilled PBS. After centrifugation, supernatant was discarded and pellet was resuspended with 100 μl of cell lysis buffer (BioVision). Following incubation for 10 min on ice, the cell lysates were centrifugated for 1 min at 10,000 × *g* and the resulting supernatant was transferred to fresh tubes on ice. The reaction was prepared in opaque-walled 96-well plates. 50 μg protein sample in a volume of 50 μl was added to the well. 50 μl of 2× reaction buffer (BioVision) containing 10 mM DTT was then added to each sample. Subsequently, VDVAD-AMC was added to the samples and incubated at 37 °C for 2 h. The sample fluorescence was measured using a fluorescence plate reader and data was expressed as percentage of caspase-2 activity over control.

### 2.9 Determination of caspase-3 proteolytic activity

Cells were treated in an opaque-walled 96-well plate at a density of 1 × 10^6^ cells per well in a volume of 200 μl and kept under 5% CO_2_ at 37 °C for 4 h. Caspase-Glo^®^ Buffer (Promega) was thawed and equilibrated to room temperature and the solution was used to dissolve lyophilized Caspase-Glo^®^ Substrate. Then, the mixture was vortexed to obtain a homogenous solution. The 96-well plate was equilibrated to room temperature for 30 min before the end of treatment. 100 μl of cell suspension was discarded from each well before 100 μl of Caspase-Glo^®^ Reagent was added to cells. The plate was placed on an orbital shaker for 30 s and then further incubated at room temperature for 2 h. The luminescence signal was then measured using a luminometer and data was expressed as percentage of caspase-3 activity over control.

### 2.10 MTT assay

Cytotoxicity of GTN was evaluated using the MTT assay. Briefly, cells were treated at 1 × 10^6^ cells/ml with final concentrations of 12.5 μM, 25 μM, 50 μM and 100 μM GTN. After 24 h incubation, 20 μl of 5 mg/ml MTT solution was added to each well and further incubated for 4 h at 37 °C. Subsequently, 150 μl medium was discarded from each well before adding 150 μl DMSO (Fischer Scientific). For complete dissolution, the plate was incubated for 15 min with gentle shaking for 5 min. The cytotoxic effects of GTN were monitored by measuring the absorbance of each well at 570 nm. Mean absorbance for each GTN concentration was expressed as a percentage of absorbance in the vehicle control wells. IC_50_ represents the GTN concentration that reduced the mean absorbance at 570 nm to 50% of those in the vehicle control wells.

### 2.11 Topoisomerase IIα-mediated DNA cleavage

Effects of GTN on stabilization of topoisomerase II-DNA complexes were evaluated using the method of Gantchev and Hunting (1997). Reactions were performed in a buffer containing 10 mM Tris–HCl, pH 7.7, 50 mM KCl, 0.1 mM EDTA, 5 mM MgCl_2_.2H_2_O, 5 mg BSA and 0.5 mM ATP. Topoisomerase II enzyme, GTN, and etoposide as a positive control, at the indicated concentrations were incubated in the buffer on ice for 10 min. Reactions were initiated by the addition of pBR322 plasmid DNA and transferred to a 37 °C water bath. Reactions contained 6 units (∼13 nM) of human topoisomerase IIα (TopoGen) and 300 ng of pBR322 DNA (New England Biolabs) per 20 μl sample volume. Following 10 min incubation, the enzyme activity was terminated and cleavage products were trapped by the addition of 2 μl of 10% SDS followed by the addition of EDTA and NaCl to 10 mM and 20 mM, respectively. Reactions were incubated for an additional 5 min at 37 °C. Enzyme trapped in cleavable complexes was removed by proteolysis with proteinase K at a final concentration of 0.8 mg/ml and incubated at 55 °C for 2 h to reveal any linearized DNA products. Samples were then electrophoresed at 2 V/cm for 18 h on 1.3% agarose gels in Tris–acetate–EDTA buffer containing 0.7 μg/ml of ethidium bromide. Using this system, different forms of migrated DNA were as follows: relaxed (greatest mobility), supercoiled, linear and nicked open circular (lower mobility).

### 2.12 Statistical analysis

The data are expressed as the mean ± standard error of mean (SEM) from at least three independent experiments. Statistical significance was evaluated using the Student's *t*-test for comparisons between two mean values whereas one-way ANOVA with Tukey post hoc test was used to test the significance between multiple groups. Differences were considered significant with a probability level of *p* < 0.05.

## 3 Results

### 3.1 Role of oxidative stress in GTN-induced apoptosis

**Fig. 1:**
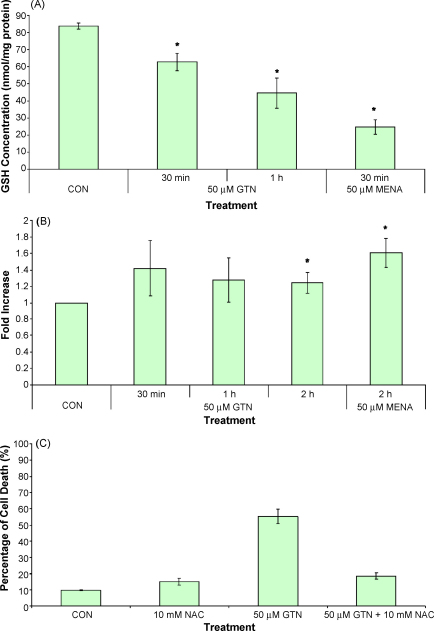
GTN induces oxidative stress in Jurkat cells. The early decrease of GSH was assessed in 50 μM GTN-treated cells at 30 min and 1 h using the Ellman reagent (A) whereas the formation of superoxide was evaluated using hydroethidine in conjunction with flow cytometry (B). Data represent the mean ± SEM from at least three independent experiments. *p < 0.05 against control.

It has been shown previously that GTN induces redox changes in MDA-MB-231 (Chen et al., 2005). In this study, Jurkat cells treated with 50 μM of GTN resulted in a significant loss of GSH as early as 30 min (Fig. 1A). This result suggests the generation of oxidative stress in these cells and further studies were carried out to monitor the production of reactive oxygen species (ROS) using the fluorescent probe hydroethidine (HE) in conjunction with flow cytometry. Our study demonstrated the ability of Jurkat cells to produce ROS when the positive control menadione was used (Fig. 1B). When Jurkat cells were treated with GTN, an increase of ethidine production, the oxidised product of HE was observed as early as 30 min consistent with the early loss of GSH (Fig. 1B). ROS production remained elevated for the first 2 h. To verify the role of ROS in GTN treatment, Jurkat cells were pre-treated with NAC, an antioxidant, and cell death was assessed as shown in Fig. 1C. Cell death induced by GTN was inhibited by NAC, further confirming the role of ROS in cytotoxic effects of GTN.

### 3.2 Early DNA damage occurs in GTN-treated Jurkat cells

**Fig. 2:**
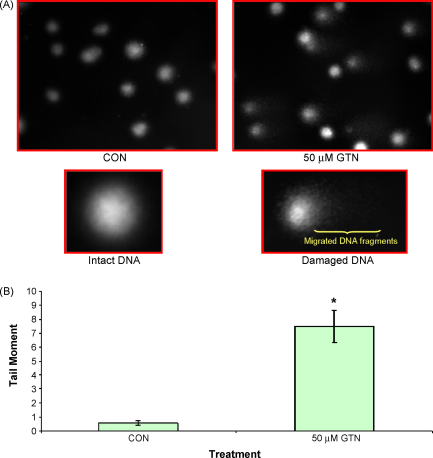
DNA damage in GTN-treated Jurkat cells. Cells were treated with 50 μM GTN for 30 min and DNA damage was assessed using alkaline comet assay as described in Section 2. A pictorial image of intact and damaged DNA in Jurkat cells is presented in panel A. The DNA damage data were expressed as tail moment (B). Data represent the mean ± SEM from at least three independent experiments. *p < 0.05 against control.

The importance of radical induced DNA damage is becoming more apparent especially with superoxide anions, hydrogen peroxide and hydroxyl radicals (reviewed in Genestra, 2007). Here, assessment of DNA damage was carried out using alkaline Comet assay. Control untreated cells show a round and healthy looking nucleus where the DNA is contained in the nucleus (Fig. 2A). In the presence of DNA damage, there will be fragments of DNA migrating out of the nucleus forming a comet-like tail. Consistent with the loss of GSH and production of ROS, GTN-treated Jurkat cells also resulted in DNA damage as measured by tail moment (Fig. 2B) as early as 30 min, which can be seen before any morphological changes associated with late apoptosis such as nuclear condensation.

**Fig. 3:**
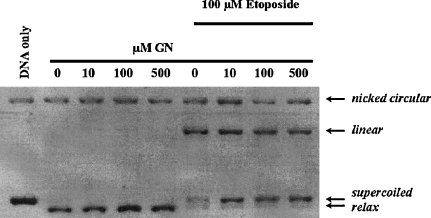
GTN induces DNA damage independent of topoisomerase II α inhibition. The effect of GTN on topoisomerase II inhibition was assessed using pBR322 DNA as described in Section 2.

In order to gain further insight into the possible nuclear target of GTN, we investigated its effects on the activity of human topoisomerase IIα (topo II) since another styryl-lactone, namely Howiinol A, is a topoisomerase II poison (Xu and He, 1999). As shown in Fig. 3, the effects of different concentrations of GTN on topo II processing of supercoiled pBR 322 DNA were determined. Increasing concentrations of GTN had no effect on the ability of topo II to relax supercoiled DNA. Etoposide, a well-known topoisomerase inhibitor (Hande, 2006) alone stabilized the enzyme-linked DNA complexes (indicated by the linear band). However, co-incubation with increasing GTN concentrations had no effect on etoposide linear band formation.

### 3.3 Caspase-2 is significantly increased but not crucial for GTN-induced apoptosis

**Fig. 4:**
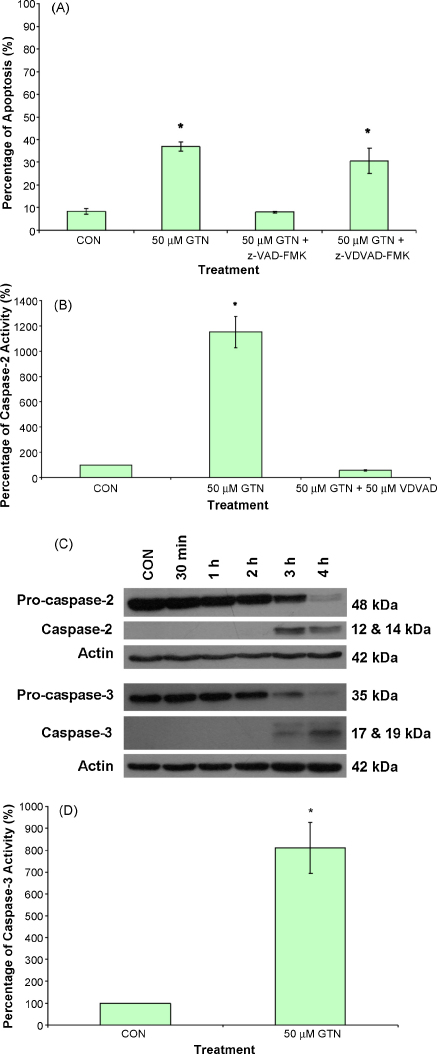
GTN-induced Jurkat cells involves caspases. Apoptosis induced by 50 μM GTN was assessed based on the externalization of phosphatidylserine (A). In some experiments the cells were pre-treated for 1 h with 50 μM Z-VAD-FMK or 50 μM Z-VDVAD-FMK before treatment with GTN for 4 h. The caspase-2 activity in GTN-treated cells was determined using specific substrate VDVAD-AMC (B). Processing of pro-caspase-2 and pro-caspase-3 in 50 μM GTN-treated cells were detected using immunoblotting (C). The caspase-3 activity was confirmed using specific caspase-3 substrate DEVD-aminoluciferin (D). Values were mean ± SEM from at least three independent experiments. *p < 0.05 against control.

It has been reported that genotoxic stress causes activation of caspase-2 upstream of mitochondria and that this caspase is the apical caspase which is required for apoptosis (Zhivotovsky and Orrenius, 2005). To determine the functional importance of apical caspase-2 during apoptosis, Jurkat cells were pre-treated with 50 μM Z-VDVAD-FMK for 30 min and apoptosis was assessed by flow cytometry using annexin V and propidium iodide. Treatment with 50 μM GTN resulted in 36.9 ± 2.0% apoptosis in Jurkat cells as shown in Fig. 4A. The specific inhibitor of caspase-2 did not block apoptosis induced by GTN even though caspase-2 proteolytic activity was completely abrogated in the presence of this inhibitor. In agreement with our previous study (Inayat-Hussain et al., 1999), the pan-caspase inhibitor z-VAD-FMK abolished GTN-induced apoptosis.

To further investigate if caspase-2 was activated in GTN-induced apoptosis, the specific caspase-2 fluorogenic substrate VDVAD-AFC cleavage activity was analysed. Cleavage of this fluorogenic substrate increased significantly to 12-fold over control in Jurkat cells after 4 h of treatment with GTN (Fig. 4B). Pre-treatment with 50 μM Z-VDVAD-FMK blocked caspase-2 proteolytic activity in GTN-treated Jurkat cells. To verify that caspase-2 processing occurred in GTN-treated Jurkat cells, Western blotting was carried out and using a specific antibody to caspase-2, we found that cleavage of this caspase to its active subunit (33 kDa) occurred as early as 3 h. The downstream executioner caspase-3 was concomitantly processed as shown in Fig. 4C. Intact caspase-3 was cleaved to its active subunit as early as 3 h and by 4 h all the intact protein was cleaved to its active subunit (17 kDa). Using a luminogenic substrate for caspase-3, an 8-fold increase of the activity of this caspase over controls was observed (Fig. 4D).

### 3.4 Bcl-2 is not crucial in GTN-induced cytotoxicity

**Fig. 5:**
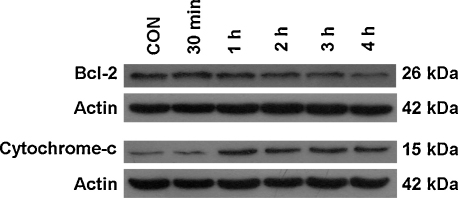
Effects of GTN on Bcl-2 expression and cytochrome *c* release in Jurkat cells. Cells were treated with various exposure times and the expression of Bcl-2 and cytosolic cytochrome *c* was detected using immunoblotting.

During apoptosis, opening of the permeability transition pore causes the release of proapoptotic proteins including cytochrome *c* which is tightly controlled by Bcl-2 family proteins (Gogvadze et al., 2008). As shown in Fig. 5, GTN treatment caused a release of cytochrome *c* as early as 1 h, however the protein level of Bcl-2 remained the same throughout the first 3 h of GTN treatment. Although there was a reduction in the level of Bcl-2 protein at 4 h, our data suggests that the reduction in Bcl-2 is not important for cell death induced by GTN.

**Fig. 6:**
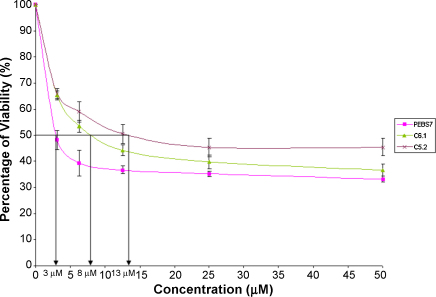
Sensitivity of Jurkat Bcl-2 stable transfectants (clones C5.2 and C6.1) to etoposide. Cytotoxicity of etoposide was assessed by MTT on vector control (pEBS7), C6.1 and C5.2 cells and the IC_50_s were 3 μM, 8 μM and 13 μM respectively.

To confirm that the anti-apoptotic Bcl-2 protein does not regulate cell death induced by GTN, a panel of Bcl-2 overexpressing Jurkat cells was employed in this study. The MTT cytotoxicity assay was employed and the inhibition concentrations (IC_50_) of GTN on the Bcl-2 stably transfected Jurkat cells were determined as shown in Table 1. Four clones of stably transfected Jurkat cells were used as described previously (Johnson et al., 1999). These clones namely J-Bcl2/C4.1, J-Bcl2/C5.2, J-Bcl/C6.1, and J-Bcl2/A8 harbor the full-length human Bcl-2 cDNA and express between a 3-7 fold increase in the Bcl-2 protein. The IC_50_ for pEBS7 vector control cells after 24 h treatment with GTN was 35 μM. Our study shows that the IC_50_s for GTN in the Bcl-2 stable transfectants ranged from 26 μM to 33 μM and were not significantly different from the control values, confirming that the oncoprotein Bcl-2 did not confer resistance to GTN cytotoxicity. We have previously shown that these panels of Bcl-2 transfectants are resistant to Fas and staurosporine (Johnson et al., 1999). Using 2 clones namely C5.2 and C6.1, we again confirmed that these BCL-2 tranfectants were resistant to another positive control, etoposide, as shown in Fig. 6.

## 4 Discussion

Styryl-lactones have gained a considerable interest in the anticancer drug development area. Currently approximately 100 compounds have been isolated and/or synthesised (de Fátima et al., 2006). In this study, the mechanism of apoptosis induced by goniothalamin was investigated. Our data show that there was a reduction of GSH as early as 30 min in GTN-treated Jurkat cells. This is consistent with a recent study where GTN-treated MDA-MB-431 cells reduced the total contents of intracellular GSH and protein thiol as assessed by monobromobimane (Chen et al., 2005). This reduction of GSH could be attributed to the production of reactive oxygen species or direct alkylation of GTN to thiol groups on proteins including GSH. Using hydroethidine in conjunction with flow cytometry, our results confirmed the production of ROS as early as 30 min (Fig. 1B). The role of ROS in GTN toxicity was confirmed using NAC in Jurkat cells (Fig. 1C) and this is in agreement with previous studies where GTN-induced apoptosis occurs via ROS in MDA-MB-231 cells, an event that is also blocked by NAC (Chen et al., 2005). This reactive oxygen species may contribute to the loss of GSH and also to DNA strand breaks, which we have shown to be independent of topoisomerase II inhibition (Fig. 3).

The DNA damage seen in GTN-treated Jurkat cells subsequently leads to apoptosis induction. Although there is no direct evidence to corroborate that DNA damage is responsible for apoptosis, our recent studies demonstrated that using mouse W7.2 T cells overexpressed with full-length receptor for activated protein kinase-1 (RACK-1), early DNA damage induced by GTN was inhibited and there was no further cytotoxicity observed (Inayat-Hussain et al., 2009). In addition to the absence of Bax, Jurkat cells also lack p53 protein expression (Karpinich et al., 2006). Therefore, the DNA damage induced by GTN resulted in p53-independent apoptosis. However, the p73 gene may replace p53 as an apoptosis signal in these Jurkat cells. Considering that p73 has p53-like properties, it is likely to be involved in DNA damage induced apoptosis in GTN-treated Jurkat cells. Several lines of evidence suggest that p73 is able to induce apoptosis in p53-independent manner. For example, overexpression of p73 or DNA damage-mediated p73 upregulation induces apoptosis in p53 null cells (Ozaki and Nakagawara, 2005). In Jurkat cells, DNA damaging agents, such as etoposide, have been demonstrated to trigger the upregulation of p73 as an apoptotic signal followed by mitochondrial translocation of truncated Bid and release of cytochrome *c* into cytosol (Karpinich et al., 2006). Due to its structural similarities to p53 protein, p73 can bind to p53-responsive elements and transactivate an overlapping set of p53-target genes such as PIDD (Ozaki and Nakagawara, 2005). The upregulated PIDD binds to RAIDD and caspase-2 forming a complex called the PIDDosome, which resulted in the processing of caspase-2, as seen in our apoptotic model.

The increase in caspase-2 activity is not crucial in GTN-induced apoptosis as a specific caspase-2 inhibitor did not abrogate the externalization of phosphatidylserine. It should be noted that caspase inhibitor blocks activity of the caspase and not the processing of its zymogen to its active form. This is important since Robertson et al. (2004) have demonstrated that processed caspase-2 can induce the release of cytochrome *c* independently of the protease activity. Furthermore, the release of cytochrome *c* precedes caspase-2 activation in GTN-induced Jurkat apoptosis as shown in Fig. 5 which is in contrast with previous findings. It has been reported that caspase-2 is activated early in the apoptotic process and induces the release of cytochrome *c* upon mitochondrial translocation (Zhivotovsky and Orrenius, 2005). Therefore, the release of cytochrome *c* in this study may well be initiated by the upregulation of p73 tumor suppressor protein (Melino et al., 2004). In addition, the release of cytochrome *c* in GTN-treated Jurkat can also be a direct effect of an early increase in superoxide level. The oxidation of critical thiol residues of VDAC and ANT has been demonstrated to cause permeabilization of the mitochondria membrane (Costantini et al., 2000; Madesh and Hajnóczky, 2001). Oxidation of mitochondrial cardiolipin releases bound cytochrome *c* into the mitochondrial intermembrane space. These apoptogenic proteins will then be release into the cytosol via exit conduit formed by oxidised VDAC and ANT pores. Once in the cytosol, the cytochrome *c* forms the apoptosome complex which activates caspase-9 in Jurkat as reported previously (Inayat-Hussain et al., 2003).

We have employed Bcl-2-overexpressing Jurkat cells to demonstrate the regulation of Bcl-2 in the release of cytochrome *c*. Intriguingly, the overexpressed Bcl-2 did not protect the cells from the cytotoxic effects of GTN as assessed by MTT assay. This was in agreement with previous studies where Bcl-2 did not confer any protection in superoxide-mediated oxidation of VDAC and ANT which resulted in mitochondrial release of cytochrome *c* (Costantini et al., 2000; Madesh and Hajnóczky, 2001). Although GTN induces a loss of Bcl-2 in wild type Jurkat, this may not be crucial as the loss is most probably due to the activation of caspase-3 which cleaves the anti-apoptotic protein as a relatively late event in apoptosis (Kirsch et al., 1999). However, the possible involvement of other members of the Bcl-2 family cannot be ruled out. The precise role of Bcl-2 in the regulation of mitochondria permeabilization is still unclear. In this respect, it is tempting to speculate that in this study, superoxide-mediated VDAC and/or ANT oxidation may disrupt the binding of Bcl-2 to these proteins. Bcl-2 may then be degraded in the cytosol via the proteosomal degradation system.

In conclusion, our data demonstrate that GTN induces ROS and DNA damage in Jurkat cells leading to the activation of an intrinsic apoptotic pathway which is independent of caspase-2 and Bcl-2 regulation.

## Conflict of interest statement

None.

## Figures and Tables

**Table 1:** Effects of GTN on a panel of Bcl-2 overexpressed Jurkat cells. Protein expression of Bcl-2 in the Jurkat clones was A8 > C5.2 > C4.1 > C6.1 with pEBS7 as vector control. Cytotoxicity of GTN on these cells was evaluated using MTT assay.

Clone	IC_50_
pEBS7	38.3 ± 6.5
C6.1	33.5 ± 13.6
C4.1	28.0 ± 8.2
C5.2	32.5 ± 2.5
A8	30.9 ± 5.2
